# Generalized anxiety disorder and resilience during the COVID-19 pandemic: evidence from China during the early rapid outbreak

**DOI:** 10.1186/s12889-021-11877-4

**Published:** 2021-10-09

**Authors:** Hao Chen, Junling Gao, Junming Dai, Yimeng Mao, Yi Wang, Suhong Chen, Qianyi Xiao, Yingnan Jia, Pinpin Zheng, Hua Fu

**Affiliations:** Preventive Medicine and Health Education Department, School of Public Health, Fudan University, No.138 Yixueyuan Road, Shanghai, 200032 P.R. China

**Keywords:** COVID-19 pandemic, Generalized anxiety disorder, Worry factor, Resilience

## Abstract

**Background:**

Generalized Anxiety Disorder (GAD) is a common but urgent mental health problem during disease outbreaks. Resilience buffers against the negative impacts of life stressors on common internalizing psychopathology such as GAD. This study assesses the prevalence of GAD and examines the protective or compensatory effect of resilience against worry factors during the COVID-19 outbreak.

**Methods:**

A cross-sectional online survey was conducted among Chinese citizens aged ≥18 years from January 31 to February 2, 2020. A total of 4827 participants across 31 provinces and autonomous regions of the mainland of China participated in this study. The Generalized Anxiety Disorder scale (GAD-7), the Connor-Davidson Resilience Scale (CD-RISC), and a self-designed worry questionnaire were used to asses anxiety disorder prevalence, resilience level, and anxiety risk factors. Multivariable logistic regression was used to identify the associations of resilience and worry factors with GAD prevalence after controlling for other covariates.

**Results:**

The prevalence of anxiety disorder was 22.6% across the 31 areas, and the highest prevalence was 35.4% in Hubei province. After controlling for covariates, the results suggested a higher GAD prevalence among participants who were worried about themselves or family members being infected with COVID-19 (adjusted odds ratio, AOR 3.40, 95%CI 2.43–4.75), worried about difficulty obtaining masks (AOR 1.92, 95%CI 1.47–2.50), worried about difficulty of distinguishing true information (AOR 1.65, 95%CI 1.36–2.02), worried about the prognosis of COVID-19 (AOR 2.41, 95%CI 1.75–3.33), worried about delays in working (AOR 1.71, 95%CI 1.27–.31), or worried about decreased income (AOR 1.45, 95%CI 1.14–1.85) compared with those without such worries. Additionally, those with a higher resilience level had a lower prevalence of GAD (AOR 0.59, 95%CI 0.51–0.70). Resilience also showed a mediating effect, with a negative influence on worry factors and thereby a negative association with GAD prevalence.

**Conclusion:**

It may be beneficial to promote public mental health during the COVID-19 outbreak through enhancing resilience, which may buffer against adverse psychological effects from worry factors.

## Background

The 2019 coronavirus outbreak (COVID-19) was reported in late December 2019 in Wuhan, China, then spread to the majority of the world in just 2 months and was reclassified as a pandemic on March 11 by World Health Organization (WHO) [1–3]. Official Chinese statistical records stated that by August 4, 2020, a total of 84,465 people had been infected, of which 4644 had died. By the same date, 17,918,582 people have been infected with COVID-19globally, and 686,703 COVID-19-related deaths were confirmed across 193 nations [4]. Several countries continue to observe a similar outbreak to that which was observed in China in February; at that time, the number of COVID-19 infected persons rapidly increased from 24,324 to 40,235 in just 7 days, and cases were recorded in every province of mainland China as well as across 24 countries [5]. China adopted tough measures, including quarantining Wuhan on January 23, enacting travel bans,closing highway, shutting down catering companies, and restricting group dinners [6]. In succession, all 31 provinces or autonomous councils have the highest public emergency responses up to January 29 [7]. Although strong policy and pressure are beneficial to controlling the spread of the virus, travel and work restrictions, as well as worry about the outbreak, could induce anxiety, depressive disorders, and post-traumatic stress disorder, which could pose a greater hazard to some individuals than COVID-19 itself [8].

Even under exposure to panic and potentially traumatic events, not everyone develops anxiety [9]. This heterogeneity in response to stress or stimuli was found to be partially owed to the protective psychological capacity of resilience, which can help people avoid psychopathology or maintain favorable mental status in the aftermath of trauma [10]. Resilience not only protects individuals from risk factors but also helps them recover or bounce back from an altered environment following an adversity [11]. The mechanisms of the promotion of mental health by resilience are recognized as facilitating intrapersonal competence (i.e., perceived competence, coping skills, and self-efficacy) and utilizing external resources appropriately (i.e., seeking friends or public agencies for help) [12]. Resilience buffers against the negative impacts of life stressors on common internalizing psychopathology such as generalized anxiety disorder (GAD) symptoms [13, 14]. During the coronavirus pandemic, GAD is unavoidable. About 41 and 23.1% of the general population of Cyprus reported symptoms associated with mild and moderate-to-severe GAD [15]. Among 1311 community-dwelling individuals in Bangladesh, 37.3% showed GAD symptoms, compared to an estimate 28% before the outbreak [16]. Risk factors for GAD problems have been described in prior research. Demographic factors predicting generalized anxiety include being female, being older (more than 30 years), having a higher education (above a bachelor’s degree), being married, and being a non-governmental employee [16]. Some behaviors like frequent social media exposure, and spending too much time thinking about the outbreak were positively associated with GAD in Chinese community adults [17, 18]. However, there has been little research on the ability of resilience, as a positive psychological factor, to protect mental health or compensate for adverse factors during a virus pandemic [19, 20]. Unlike the deficit model, resilience in positive psychology means more than simply the absence of mental disorder, but functions as a reserve or intrinsic capacity that can be drawn on as a buffer against a wide range of future adversities [21, 22]. Psychological intervention with resilience promotion is suitable for public mental health and proved effective to the general population during the COVID-19 pandemic [23]. In this study, we conducted a survey to assess anxiety among a Chinese population and examine the potential risks and protective effect of resilience for GAD in early rapid outbreak periods.

## Methods

### Study design and participants

From January 31 to February 22,020, we recruited a sample of 5851 participants aged over 18 years through an online survey and a relevant smartphone link pushed to the WeChat ‘moment circle’ (a function that can share personal photo or public website link in your moment which is visible to friends on platforms like Twitter and Facebook) by the researches or participants who volunteered to pushed this questionnaire link (Wenjuanxing platform, https://www.wjx.cn/app/survey.aspx). We calculated the minimum sample size, which was 4668 participants, using the following formula: $\frac{Z^2{}_{1-\upalpha /2}p\left(1-p\right)}{d^2}$ (*z* ^ 2 (1 − α/2) =1.96; the latest prevalence of GAD (p) was 7.6% [24], d = 0.1) [25]. All of the participants were required to understand and fill out the questionnaire by themselves through their personal their WeChat account. The survey used convenience sampling to recruit suitable Chinese residents from 31 provinces and autonomous regions (China consists of a total of 34 provinces and autonomous regions) that volunteered to participate. To maximize participant motivation, respondents were entered into a drawing following completion of the questionnaires; those selected received a small monetary reward (RMB 2–100). All questionnaires took approximately 10 min to complete, and an item with a required answer was established to avoid the return of invalid questionnaires. After cleaning up invalid questionnaires (including those that were returned incomplete and those that were completed in less than 5 min), 4827 participants were included in the present study. This study was approved by the Institutional Review Board of Fudan University, School of Public Health (IRB#2020-01-0800) on January 31 and electronic consent was also given by participants by signing the first page of the survey.

### Measurements

#### Generalized anxiety disorder, Spitzer (GAD-7)

The Generalized Anxiety Disorder scale (GAD-7), developed by Spitzer, et.al, is a self-report measure that quantifies the frequency of seven symptoms (e.g., trouble relaxing, worrying too much about different things) over the past 2 weeks to screen for anxiety disorder [26]. Responders are asked to rate each item on a Likert scale from 0 (“not at all”) to 3 (“nearly every day”); the items are summed for an overall score ranging from 0 to 21, with higher scores reflecting more severe GAD symptoms. The Chinese version of the GAD-7 (C-GAD-7) has been demonstrated to have acceptable reliability and validity [27]. The pervious normative study defines degree of anxiety into four categories with three cut-offs: no (0–4), mild (5–9), moderate (10–14), and severe anxiety (≥15) [26, 28]. Considering evidence that some level of anxiety is normal during the COVID-19 pandemic, a scale cut-off of 10 and above to represent GAD is appropriate [29].

#### Resilience, Connor Davidson (CD-RISC-10)

The abridged Connor-Davidson Resilience Scale (CD-RISC) is a self-administered, 10 item scale that reflects the ability to tolerate and overcome adverse situations such as illness, pressure, and failure (item examples: “Tend to bounce back after illness or hardship” and “Can stay focused under pressure”) [30]. Each item is rated on a 5-point Likert scale, ranging from 0 (“not true at all”) to 4 (“true nearly all the time”), and a higher total score indicates greater resilience. The reliability coefficient of the Chinese version of the CD-RISC was found to be 0.91 [31]. Due to the lack of a recognized cut-off point, resilience scores are usually categorized into three groups: high resilience (score ≥ 75th percentile), medium resilience (score < 75th percentile and > 25th percentile), and low resilience (score ≤ 25th percentile) [32].

#### Worry factors to COVID-19

Our self-designed worry questionnaire consisted of six items following the question, “Have you been bothered or worried by the following recently?” Respondents were asked to rate each question on a Likert scale from 1 (“not worried at all”) to 5 (“very worried”). The questions were categorized into three dimensions and selected from prior research: 1) perception of susceptibility to COVID-19 (1 question), i.e., worry about COVID-19 infection in oneself or family members [33]; 2) perceived barriers to preventing COVID-19 (2 questions), i.e., worry about difficulty obtaining safety equipment such as medical masks or worry about difficulty distinguishing authentic [34] and valid information about the COVID-19 pandemic across various social media platforms [35]; 3) perceived hazard of COVID-19 (3 questions), i.e., worry about the prognosis of COVID-19 [36], delays in working, or decreased income [37]. In the present study, the Cronbach’s alpha coefficient for the internal consistency (reliability) of worry factors to COVID-19 was 0.81 and two subscale were 0.77 (perceived barriers to preventing COVID-19) and 0.63 (perceived hazard of COVID-19). However, perception of susceptibility to COVID-19 was not suitable to calculate individual reliability for only containing one item. The sampling adequacy for the 6-item scale was excellent (Kaiser-Meyer-Olkin = 0.91). Inter-item correlations were sufficiently large for principal components analysis (PCA) (Bartlett’s test of sphericity: *χ*^2^ (21) =8299.1, *p* < 0.001). The PCA revealed three factors, which in combination explained 72.10% of the variance. An examination of the factor loadings after rotation suggested as expected that factor 1 (perceived hazard of COVID-19) had three items (loading factors, namely, worried about prognosis (0.67), working delay (0.79), and decreased income (0.81); factor 2 included 2 items, difficulty obtaining safety equipment (0.79) and distinguishing authentic health information (0.78); factor 3 only contained 1item, worried about susceptibility to COVID-19 (0.86). The three factors accounted for 33.83, 22.16, and 16.11% of the explained variance, respectively.

### Covariates

Covariates in this study included sex, age, educational level [junior college (education for 16–18 year old and not award academic degree) and above (junior college, bachelor (education for > 18 year old), master and doctor), under junior college (junior high school, senior high school)], marital status [married, unmarried and other (including divorced and widowed)], areas (Hubei province, cities with COVID-19 ≤ 100, and cities with COVID-19 > 100), location (city, town and village), and community COVID-19 pandemic status, measured by questions such a “Are there COVID-19 patients, medical observations or suspected patients in your living community/neighborhood?” (no COVID-19 cases, under medical observation, suspected cases, confirmed cases, unknown,) and ones regarding exposure to Hubei within the past month (yes, no,) and whether the participants had medical workers or people with a medical education background in their family (yes, no).

### Statistical analysis

The demographic characteristics and exposure history of the study sample are presented as means with *SD*s or percentages by different levels of COVID-19 outbreak. Based on the COVID-19 outbreak level of every province (latest data from February 1, 0:00 to 24:00) [38], we divided these 31 areas into three subgroups—Hubei, with > 10,000 COVID-19 patients, 16 provinces or auto regions with ≥100 & ≤10,000 COVID-19 patients, and 14 provinces or autonomous regions with < 100 COVID-19 patients. The *χ*^2^ test was used to examine the distribution differences in GAD prevalence, anxiety worry factors, and resilience among the above three areas. Multivariable logistic regression analyses were used to explain the associations among the prevalence of GAD, worry factors, and resilience after controlling for covariates. We considered two-sided *p*-values of less than 0.05 statistically significant. All of the analyses were performed using SPSS version 22.0 (SPSS, Chicago, IL, USA). Structural equation modeling was used to assess the standardized coefficients (SSCs) among resilience, GAD, and anxiety risk factors. Amos 22.0 (SPSS, Chicago, IL, USA) was used to determine whether the data fit the model.

## Results

### Demographic characteristics and exposure history of participants

Our analysis including 4827 participants dispersed across 31 provinces and autonomous regions in mainland of China. They were aged between 18 and 85 (mean, 32.32; SD, 9.98); 67.7% of them were female, and 62.5% were urban residents. 78.5% participants had received bachelor or master education. 36.7% of participants were still working and 20.8% were students. 54.0% of them were married. 76.8% participants reported non-confirmed or related cases exposure in their living community, while 8.4% participants reported under medical observation cases exposure, suspected cases (5.4%), confirmed cases (9.4%) in their living neighborhood. Only 4.9% participants living in Hubei and 7.9% had travelled to Hubei since November, 2019.

### GAD prevalence and distribution of worry factors to COVID-19

According to cut-off points, 32.7% of respondents had mild GAD (score: 5–9), 13.0% had moderate GAD (score: 10–14), and 9.6% had severe GAD (score: ≥15). In the present study, we selected a GAD scale cut-off point of 10 and found that the prevalence of GAD was 22.6% among the respondents. Of participants, 37.5% reported medical education background in their immediate family, while 78.9% of participants reported worry about the outbreaks regarding susceptibility to COVID-19, difficulty obtaining masks (72.6%), difficulty distinguishing valid information (75.0%), decreased income (79.4%), delays in working, and worry about the prognosis of COVID-19 (85.9%). As shown in Table 1, univariate analysis indicated that GAD prevalence, worry about susceptibility to COVID-19, worry about barriers to preventing COVID-19, and worry about the hazards of COVID-19 were higher in Hubei than in the other affected areas (*P* < 0.05).

**Table 1:** The detailed distributions of areas with different outbreak levels

	Total	COVID-19 > 10,000 in Hubei province, *n* = 130	COVID-19 ≥ 100 & ≤10,000 in 16 provinces or autonomous regions, *n* = 3464	COVID-19 < 100 in 14 provinces or autonomous regions, *n* = 1233
Age (years, mean (standard deviation))	32.3 (9.9)	29.9 (9.2)	32.2 (9.9)	32.9 (10.1)
Male	1560 (32.3%)	54 (41.5%)	1104 (31.9%)	402 (32.6%)
Location				
City	3018 (62.5%)	71 (54.6%)	2152 (62.1%)	795 (64.5%)
Town	902 (18.7%)	28 (21.5%)	651 (18.8%)	223 (18.1%)
Village	907 (18.8%)	31 (23.8%)	661 (19.1%)	215 (17.4%)
Bachelor degree and above	3778 (78.5%)	108 (83.0%)	2742 (79.2%)	938 (76.1%)
Community COVID-19 epidemic				
Non COVID-19 case	3194 (66.2%)	47 (36.2%)	2282 (65.9%)	865 (70.2%)
Under medical observation	404 (8.4%)	9 (6.9%)	304 (8.8%)	91 (7.4%)
Suspected case	262 (5.4%)	18 (13.8%)	178 (5.1%)	66 (5.4%)
Confirmed case	454 (9.4%)	31 (23.8%)	302 (8.7%)	121 (9.8%)
Unknown	513 (10.6%)	25 (19.2%)	398 (11.5%)	90 (7.3%)
Exposure to Hubei during a month	266 (5.5%)	–	208 (6.0%)	58 (4.7%)
Medical worker in your family	1812 (37.5%)	42 (32.3%)	1276 (36.8%)	494 (40.1%)
GAD prevalence	1090 (22.6%)	46 (35.4%)	754 (21.8%)	290 (23.5%)
High resilience	3582 (74.2%)	95 (73.1%)	2572 (74.2%)	915 (74.2%)
Worried self and family member were susceptibility to COVID-19***	3810 (78.9%)	114 (87.7%)	2739 (79.1%)	957 (77.6%)
Worried about difficulty obtaining masks***	3672 (76.1%)	105 (80.8%)	2647 (76.4%)	920 (74.6%)
Worried about difficulty distinguishing valid information***	3503 (72.6%)	111 (85.4%)	2531 (73.1%)	861 (69.8%)
Worried about decrease income***	3618 (75.0%)	104 (80.0%)	2609 (75.3%)	905 (73.4%)
Worried about delay working***	3833 (79.4%)	114 (87.7%)	2766 (79.8%)	953 (77.3%)
Worried about prognosis of COVID-19	4148 (85.9%)	111 (85.4%)	2963 (85.5%)	1074 (87.1%)

*** representing *P* value < 0.001

### Worry factors to COVID-19, resilience, and GAD prevalence

The crude associations among sources of anxiety disorder, resilience, and GAD prevalence are shown in Model 1, Table 2. The medium and high resilience groups were combined into a high resilience group due to the lack of statistical difference between the two groups (*P* > 0.05). Those with a higher resilience level had a decreased GAD prevalence (OR 0.65, 95%CI 0.56–0.76). Our findings also showed a higher GAD prevalence among participants who worried about themselves or family members being infected with COVID-19 (OR 3.12, 95%CI 2.25–4.34), difficulty obtaining masks (OR 1.75, 95%CI 1.35–2.26), difficulty distinguishing information (OR 1.83, 95%CI 1.51–2.22), the prognosis of COVID-19 (OR 2.37, 95%CI 1.72–3.26), delays in working (OR 1.70, 95%CI 1.27–2.27), or decrease income (OR 1.62, 95%CI 1.28–2.01) compared to those without such worries. Similar results regarding resilience and GAD risk factors were found in Model 2. We also found that participants with higher educational attainment (bachelor degree and above) had lower GAD prevalence (AOR 0.56, 95%CI 0.47–0.67), while those with exposure to Hubei province in the past month showed an increased GAD prevalence (AOR 1.90, 95%CI 1.42–2.54).

**Table 2:** Odds ratios for GAD prevalence by demographic characteristic, resilience and worried factors to COVID-19

	Cases	Model 1	Model 2
		OR (95%CI)	OR (95%CI)
Age (years)			
18–24	152 (20.6%)		1(ref)
25–44	734 (25.7%)		1.43 (1.14,1.79)
45 and above	204 (16.6%)		1.09 (0.79,1.49)
Sex			
Male	359 (23.0%)		1(ref)
Female	731 (22.4%)		0.90 (0.77,1.06)
Marriage status			
Unmarried	375 (17.8%)		1(ref)
Married	676 (25.9%)		1.52 (1.24,1.86)
Divorced or widowed	37 (33.0%)		2.32 (1.44,3.75)
Education attainment			
Below college	347 (33.4%)		1(ref)
College and above	743 (19.6%)		0.56 (0.47,0.67)
Location			
City	683 (22.6%)		1(ref)
Town	200 (22.2%)		0.82 (0.67,1.00)
Village	207 (22.8%)		0.91 (0.74,1.11)
Cities with different COVID-19 prevalence			
Cities of COVID-19 < 100	290 (23.5%)		1(ref)
Cities of COVID-19 > 100	754 (21.8%)		0.89 (0.76,1.06)
Hubei (COVID-19 > 10,000)	46 (35.4%)		0.99 (0.61,1.63)
Exposure to Hubei during a month			
No	715 (21.7%)		1(ref)
Yes	127 (33.4%)		1.90 (1.42,2.54)
Medical worker in your family			
No	715 (23.7%)		1(ref)
Yes	375 (20.7%)		0.96 (0.82,1.12)
Resilience			
Lower level (1st quartile)	398 (32.0%)	1(ref)	1(ref)
Higher level (2nd to 4th quartile)	692 (19.3%)	0.65 (0.56,0.76)	0.59 (0.51,0.70)
Worry factors to COVID-19 (ref: not worry)			
Worried self and family member were susceptible to COVID-19	1042 (27.3%)	3.12 (2.25,4.34)	3.40 (2.43,4.75)
Worried about difficulty obtaining mask	1002 (27.3%)	1.75 (1.35,2.26)	1.92 (1.47,2.50)
Worried about difficulty distinguishing information	934 (26.7%)	1.83 (1 .51,2.22)	1.65 (1.36,2.02)
Worried about prognosis of COVID-19	1043 (25.1%)	2.37 (1.72,3.26)	2.41 (1.75,3.33)
Worried about delay in working	1017 (26.5%)	1.70 (1.27,2.27)	1.71 (1.27,2.31)
Worried about decreased decrease income	973 (26.9%)	1.62 (1.28,2.01)	1.45 (1.14,1.85)

*OR* Odds ratio, *AOR* Adjusted odds ratio

### The *SEM* of worry factor to COVID-19, resilience, and GAD

**Fig. 1:**
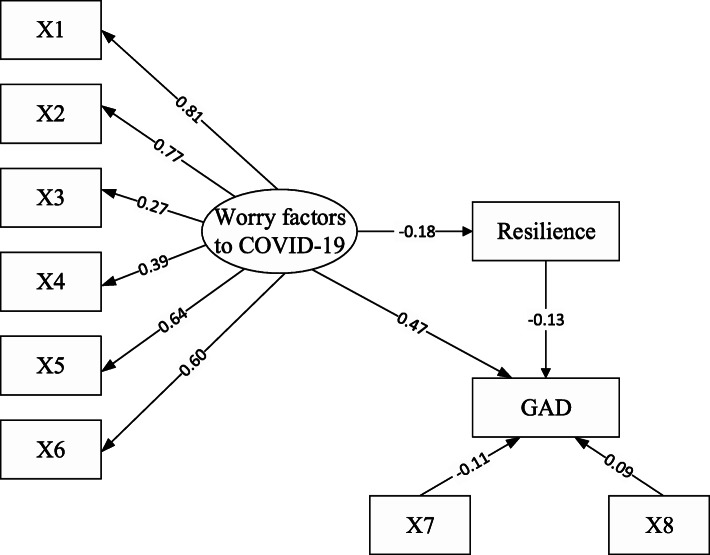
The paths among worry factors, resilience and GAD. (X1: worried self and family members were susceptible to COVID-19; X2: Worried about difficulty obtaining mask; X3: worried of obstructive to distinguish information; X4: worried about prognosis of COVID-19; X5: worried about a delay in working; X6: worries about decreased income; X7: education attainment; X8: travel to Hubei Province during the past month)

As shown in Fig. 1, structural equation modeling (SEM) was used to examine the combined underlying psychological mechanism of GAD prevalence. Compared to the criteria of goodness-of-fit statistics, SEM was a better fit to the data (*χ*^2^ degrees of freedom [df] = 168.82; root mean square error of approximation [RMSEA] = 0.04; goodness of fit index, [GFI] = 0.99; comparative fit index [CFI] = 0.99), and all of the paths were statistically significant (*p* < 0.05). Our findings suggested a mediating effect of resilience, with influenced worry factors in the negative direction (SSC = − 0.18, *p* < 0.001) and was thereby negatively associated with GAD prevalence (SSC = − 0.13, *p* < 0.001). Worry factors were also indirectly positively associated with GAD via resilience (SSCs = − 0.18 × − 0.13, *p* < 0.001) and directly associated with GAD (SSC = 0.47, *p* < 0.05). Meanwhile, high education attainment was negatively correlated with GAD (SSC = − 0.11, *p* < 0.001), and exposure to Hubei in the past month was positively associated with GAD (SSCs = 0.09, *p* < 0.05).

## Discussion

In this study, 22.6% of the sample experienced moderate to severe GAD during the early rapid outbreak in China. Hubei province, the first and worst outbreak area with over 10,000 COVID-19 patients, showed the highest GAD prevalence (35.4%). Participants who had traveled to Hubei within the past month also showed an increased GAD risk (AOR 1.90, 95%CI 1.42–2.54). This GAD prevalence during the outbreak period was much higher than that found by the latest report in a national sample, which indicated a 7.6% prevalence of anxiety disorders in China [24]. During the SARS [39], MERS [33], and Ebola [24] outbreaks, people were also more vulnerable to mental disorders caused by specific worry factors due to the high possibility of being infected by these viruses, the lack of appropriate prevention measures, and the strict biosecurity restrictions.

Determining the specific worry factors contributing to public anxiety may be beneficial to preventing mental disorders. This study found that worry about oneself or family members being infected with COVID-19 was associated with highest GAD prevalence, which is comparable to results from South Korea regarding fear of infection [33]. During the early stage of an outbreak, wearing a medical mask is one of the prevention measures recommended by WHO to limit the spread of certain respiratory diseases [40]; the initial shortage of these masks caused social panic [34]. Despite rationing of masks to each community to ensure hospitals’ demands were met, the hospitals still asked for social donations of protective supplies [34], which may cause higher GAD prevalence associated with obtaining masks in this study. While travel was restricted, people were still exposed to erroneous information on social media like prognosis of COVID-19 patients’ sequela or death and that drinking can prevent coronavirus infection, making it difficult for people to distinguish valid information [35], which may increase the GAD prevalence. Although COVID-19 vaccination opened to market for several weeks, the global inequities of vaccine distribution due to only a limited number of countries possess capacity to manufacture vaccines on their own against the virus [41].; thus, people may still be vulnerable to anxiety disorders due to concerns regarding the susceptibility and prognosis of COVID-19. Participants who were worried about delays in working and about decreased income were directly influenced by the uncertain work resumption time, for that stressful income situations can increase common mental health problems like anxiety, depression, etc. [42].

The most important finding of this study was the potential protective factors related to GAD. Resilience, the ability to maintain a state of normal equilibrium in the face of extremely unfavorable circumstances [11], has been demonstrated to have protective effects against mental disorders like depression [43], anxiety [44], and PTSD [45] after trauma exposure, which is consistent with the findings of this study. The capacity to tolerate high levels of fear and still perform efficiently within a military context has been demonstrated to be associated with high resilience [46], further explaining how high resilience is associated with low anxiety (fear of COVID-19). Additionally, we found that those with a college education or above had only about half the GAD prevalence of those with a lower education level. Considering the difficulties in distinguishing valid and truthful information, which is a source of public anxiety, more educated people can more easily find, distinguish, and accept correct information [47]. Furthermore, high educational attainment enhances one’s resilience level, which may indirectly compensate for mental disorders, especially after trauma [9, 48]. Ahmed performed a review and concluded that two underlying types of positive factors promote resilience: internal characteristics (self-esteem, trust, resourcefulness, secure attachments, sense of humor) and external factors (safety, religious affiliation, strong role models) [49]. Furthermore, this study identified a possible buffering function of resilience that can mediate the unfavorable effect of worry factors on GAD prevalence, which is consistent with previous research [50]. Moreover, the buffering effect of resilience on mental disorders caused by posttraumatic stress has been found to be stronger in individuals with more trauma experience [51]. Resilience is not a stable characteristic but a changeable one. For example, enhancing emotional regulation, cognitive flexibility and reappraisal, positive emotions, meaning-making, having purpose in life, and the ability to harness social support may help reinforce resilient functioning [52]. An asset-based approach through promoting resilience could be a novel approach to promoting public health—building upon the recognized strengths and developing the potential strengths of individuals, communities, and organizations rather than focusing on avoiding individual risk factors [53]. A study conducted at an Ottawa company using an asset mapping method identified 25 assets types that can enhance resilience levels in seven categories: (a) awareness, (b) human resources, (c) information and communication, (d) leadership and culture, (e) operational infrastructure, (f) physical resources, and (g) social capital [54]. An asset-based approach is suitable for addressing different characteristics of communities, regions, and even countries to help enact target policies.

There are some potential limitations to this study. First, causal inferences regarding the effects of worry factors and resilience on GAD prevalence cannot be made from this cross-sectional study. Second, due to the online survey process, selection bias, such as having fewer male respondents, may have affected the results. Lastly, this study did not collect data on mental disorder fluctuations that may characterize early rapid outbreak periods to some extent; the mental disorder prevalence could fluctuate over the course of the COVID-19 pandemic.

## Conclusion

In conclusion, the findings of this study suggest that there is currently a high prevalence of GAD in China, especially in the first and worst outbreak area, Hubei province, and among those who traveled to Hubei in the month prior to data collection. Furthermore, all of the worries except worry about the prognosis of COVID-19 were found to be more prevalent in Hubei Province than in other outbreak areas. Worries about susceptibility to COVID-19 are positively and highly associated with GAD prevalence, and perceived barriers to preventing COVID-19 and perceived hazards of COVID-19 are also positively associated with GAD prevalence to some extent. A high level of resilience and higher education attainment are protective factors against GAD; resilience, in particular, may buffer against the development of mental disorders due to anxiety risk factors. It may be beneficial to decrease public anxiety prevalence through health education to enhance efficacy for accessibility of medical or mask supplies and objective understanding of infectiousness, harmfulness of novel coronavirus. The resilience promotion may be a critical and conducive program for COVID-19 mental health promotion.

## Data Availability

The data that support the findings of this study are available from the School of Public Health of Fudan University apply to the availability of these data, which were used under license for the current study, and so are not publicly available. Data are however available from the authors upon reasonable request and with permission of School of Public Health of Fudan University.

## Abbreviations

CI: Confidence interval
GAD: Generalized Anxiety Disorder scale
OR: Odds ratio
SPSS: Statistical Package for the Social Sciences
USA: United States of America
